# Food-Handling Practices and Environmental Factors Associated With Food Contamination Among Street Food Vendors in Nairobi County, Kenya: A Cross-Sectional Study

**DOI:** 10.24248/EAHRJ-D-16-00382

**Published:** 2017-03-01

**Authors:** Emmah Nyambura Kariuki, Zipporah Waithera Ng’ang’a, Peter Wanzala

**Affiliations:** a Jomo Kenyatta University of Agriculture and Technology, Juja, Kenya; b Kenya Medical Research Institute, Nairobi, Kenya

## Abstract

**Background:**

Lack of adequate sanitation and refuse disposal facilities are among the factors found to contribute to food contamination among street food vendors. Most vending facilities are near crowded places, such as bus terminals or markets to attract consumers, and the few basic amenities, such as toilets, are inadequate. The objective of the study was to determine which sanitation practices were associated with food contamination in Githurai and Gikomba markets in Nairobi County.

**Methodology:**

Using a cross-sectional study design, we systematically randomly sampled 149 street food vendors and used questionnaires to interview them and make observations.

**Results:**

A significant negative association was observed between access to a toilet facility and food contamination (*P*<.001), with a decreased risk of occurrence of food contamination observed where vendors had access to a toilet facility (OR 0.095; 95% confidence interval [CI], 0.039–0.227). Accessibility of running water around the toilet facility was negatively associated with food contamination (*P*<.001), with vendors who reported access to running water having a lower occur-rence of food contamination (15.9%) compared with those who had no access to running water (30%). Presence of pests/rodents was significantly associated with food contamination (*P*<.001), with vendors who reported presence of pests/rodents having a 5.9-fold risk (OR 5.921; 95% CI, 2.831–12.383) of contaminated food. Access to fresh running water while preparing food, hand washing before handling food, and use of an apron were the food-handling practices that were negatively associated with food contamination (*P*<.005). Use of a head cover, hand washing after handling raw food, and the way food was served and stored had no statistically significant association with food contamination (*P*>.05).

**Conclusions:**

Access to a toilet facility and availability of running water within the toilet facility decreased the likelihood of food contamination. The presence of pests/rodents had a positive association with food contamination. There is a need for more basic amenities, especially toilets and water facilities, within these markets, as well as sensitisation on pest control.

## INTRODUCTION

Street-vended foods are defined as those foods prepared on the street and ready to eat, or prepared at home and consumed on the street without further preparation.^1^ Foodborne diseases are common in developing countries, including Kenya, because of the prevailing poor food-handling and sanitation practices, inadequate food safety laws, weak regulatory systems, lack of financial resources to invest in safer equipment, and lack of education for food handlers.^2^ The street food trade is a growing sector in many developing countries today, and the expansion is linked with urbanisation and the need of urban populations for both employment and food. The safety of street foods is a major consideration, which deserves and has received attention. Unsafe sources, contaminated raw food items, improper food storage, poor personal hygiene during food preparation, inadequate cooling and reheating of food items, and a prolonged time lapse between preparing and consuming food items were mentioned as contributing factors for outbreak of foodborne diseases in United States.^3^

Studies conducted in different parts of Ethiopia also showed the poor sanitary conditions of catering establishments and the presence of pathogenic organisms like *Campylobacter, Salmonella, Staphylococcus aureus, Bacillus cereus*, and *Escherichia coli*.^4–8^ Factors implicated in causing microbial contamination include poor food preparation and handling practices, inadequate storage facilities, a lack of personal hygiene among vendors, and a lack of adequate sanitation and refuse disposal facilities.^9,10^

## MATERIALS AND METHODS

### Study Area

The study area was Gikomba and Githurai markets within Nairobi County, Kenya. Two study areas were involved to expand the sample and to avoid vendors influencing each other in their responses, since vendors are closely positioned in both of the 2 markets.

Gikomba is a market located about 800 metres from the town centre in Kamukunji Constituency. Today there are more than 4,000 traders in Gikomba. Gikomba is famous for secondhand clothes sellers, but there are other products sold, including food. It is a very busy market where there are various businesses and activities such as human carriers and hand carts (*mikokoteni*) who ferry goods across the market. The surrounding communities include mainly low-income earners who largely depend on low-cost street-vended foods.

Githurai market is located in the eastern part of Nairobi, about 12 km from the city centre in Kasarani Constituency. This area has a population of over 300,000, and Githurai is a busy market famous for the sale of secondhand goods as well as food. The Githurai market is very congested, and movement within it is severely hampered as traders try to display their goods for passersby to buy. The majority of people in the surrounding communities are low-income earners who depend on the market to buy cheaply priced goods and street-vended foods. Studying food safety in these 2 areas is important as contamination in these areas may imply a possible foodborne disease outbreak that may affect a large population of those who reside in Nairobi or who visit the markets.

### Study Design

The study used a descriptive cross-sectional study design to establish the bacteriological safety of street-vended foods and assess the food-handling practices and environmental factors associated with food contamination at consumption point.

### Study Population

The study population comprised street food handlers who were selling ready-to-eat foods in Gikomba and Githurai markets. We interviewed the selected food handlers and then purchased a food sample from them for microbial analysis. The inclusion criteria for the study were street food vendors 18 years and older who gave consent. Those who were under 18 years old and who did not give consent were excluded from the study.

### Sample Size Determination

The total sample size was determined by the formula of Fisher and colleagues, where n = the desired sample size for a target population greater than 10,000; Z = normal standard deviation corresponding to 95% confidence interval (CI), that is 1.96; P = proportion of the population estimated to have desired characteristics; q=1−P; and d = degrees of accuracy desired (0.05). Hence, this study used a *P* value of 20% as used in a similar study in Ethiopia.^11^

The sample size was therefore calculated as follows:

n = [Z^2^(1−*β*)pq]/d^2^

Description:

n = required sample size
Z = confidence level at 95% (standard value of 1.96)
P = estimated prevalence (.20)
d = level of precision at 5% (0.05)
n = 245.86

However, the population under study was less than 10,000: a preliminary study done at the 2 areas revealed the population of interest was a total of 380 street food vendors. Hence the Cochran 2000 formula^12^ was further used to calculate the actual sample size.

Sample size therefore was as follows:

n_f_= n/1 + n/N
[where N = population size,
n = sample size if N is infinite (N > 10,000),and
nf = sample size if N is finite (N < 10,000)]
= 245.86/1 + 245.86/380
= 149

Then, sharing the sample proportionate to size:

Gikomba = 197/380 * 149 = 77, and
Githurai = 183/380 * 149 = 72.
Sampling interval (K) = N_1_/n_1_
= 380/149
= 2.55 ≈ 2.0

### Sampling

We used random sampling to sample the first street food vendor who was specifically preparing and selling the foods on-site within the 2 study areas, after which systematic random sampling, using a sampling interval of 2 as calculated above, was used to sample the rest of the food vendors. We then bought and aseptically collected food samples from the same street food vendors for the purpose of microbial analysis.

### Data Collection

We first distributed an informed consent form to the street food vendors to obtain consent. We then administered a structured questionnaire to gather relevant information from the street food vendors. The questionnaire included questions about food-handling practices (such as washing of hands before and after handling food, use of soap during washing of hands, washing of utensils used for food preparation, storage of foodstuffs, training on food handling, frequency of medical examination) and environmental factors (such as availability of toilet facilities, accessibility and availability of clean water near the toilet facilities, and disposal of solid waste).

We then bought food samples, which were collected aseptically in sterile universal bottles, transported to the National Public Health Laboratories under low temperature in an ice cooler box, and stored at 4°C until testing. All the samples were analysed within 24 hours of sampling. We used standard methods for enumeration, isolation, and identification of bacteria.

### Data Management and Analysis

Data from the study was first coded. Double entry was then done using Microsoft Access for comparison purposes. Errors were minimised by cleaning and rechecking all the entries with the original data forms. Data analysis was done using SPSS software (Version 20) where descriptive statistics like mean, frequencies, and percentages were used to describe the data and presentation was done through tables, pie charts, and graphs. Chi-square was used to establish the relationship between categorical independent variables (such as food-handling and environmental factors) and the dependent variable (food contamination). Correlation technique was used to establish the association between variables under study. Multivariate analysis was performed to calculate the adjusted odds ratio for the independent association between food contamination and the predictive variables.

### Ethical Considerations

Approval to conduct this study was obtained from the Scientific Steering Committee at the Kenya Medical Research Institute and the Scientific Ethical Review Committee for scientific and ethical approvals respectively. Study participants were assured that there would be no risks involved in responding to the questions and that they were free to respond or not.

The respondents were informed that the direct benefit of being involved in this study was that the information gathered would reveal some of the environmental challenges they face for the relevant parties to take action. They were also informed that the feedback from this study would assist them in understanding some of the hygienic practices they personally need to improve to prevent food contamination.

## RESULTS

Several environmental factors were significantly associated with food contamination. A negative association (*P*<.001) was observed between access to a toilet facility and food contamination, whereby a decreased risk of occurrence of food contamination (OR 0.095; 95% CI, 0.039–0.227) was observed if one had access to a toilet facility (Table 1). Vendors who had access to a toilet had a lower occurrence of food contamination (22.1%) compared with those who had no access to a toilet facility (75%) (Table 2). The vendors were further probed on the type of toilet facility they had access to, and 46.3% of the vendors had access to a modern toilet, which can be described as a pour/flush toilet, while 29.5% had access to a latrine, which is basically a deep pit that is dug for use as a toilet facility.

**TABLE 1.** Bivariate Analysis of Environmental Factors in Relation to Occurrence of Food Contamination

Environmental Factors	Chi-square	df	*P* Value	OR	Lower CI	Upper CI
Access to a toilet facility	33.598	1	<.001^*^	0.095	0.039	0.227
Type of toilet facility	37.270	2	<.001^*^	…	NA	NA
Running water within or outside the toilet facility	36.046	2	<.001^*^	…	NA	NA
Waste disposal	1.369	2	.504	…	NA	NA
Presence of pests/rodents	24.176	1	<.001^*^	5.921	2.831	12.383
Type of pests/rodents	35.489	2	<.001^*^	…	NA	NA
Environmental contaminants	2.514	1	.113	0.363	0.099	1.327

Abbreviations: CI, confidence interval; df, degrees of freedom; OR, odds ratio. Ellipses indicate the OR could not be calculated due to multiple responses to the variable.

* Variables significant at the 5% level.

**TABLE 2.** Occurrence of Food Contamination in Relation to Environmental Factors

	Food Contamination		
Environmental Factors	Yes No. (%)	No No. (%)	Total No. (%)
Access to a toilet facility			
Yes	25 (22.1)	88 (77.9)	113 (100)
No	27 (75.0)	9 (25.0)	36 (100)
**Total**	52 (34.9)	97 (65.1)	149 (100)
Type of toilet facility			
Latrine	5 (11.4)	39 (88.6)	44 (100)
Modern	20 (29.0)	49 (71.0)	69 (100)
N/A	27 (75.0)	9 (25.0)	36 (100)
**Total**	52 (34.9)	97 (65.1)	149 (100)
Running water within or outside the toilet facility			
Yes	10 (15.9)	53 (84.1)	63 (100)
No	15 (30.0)	35 (89.5)	50 (100)
N/A	27 (75.0)	9 (25.0)	36 (100)
**Total**	52 (34.9)	97 (65.1)	149 (100)
Waste disposal			
Open area	31 (33.3)	62 (66.7)	93 (100)
Municipal container	19 (40.4)	28 (59.6)	7 (100)
Other	2 (22.2)	7 (77.8)	9 (100)
**Total**	52 (34.9)	97 (65.1)	149 (100)
Presence of pests/rodents			
Yes	33 (60.0)	22 (40.0)	55 (100)
No	19 (20.2)	75 (79.8)	94 (100)
**Total**	52 (34.9)	75 (79.8)	149 (100)
Type of pests/rodents			
Rats	18 (46.2)	21 (53.8)	39 (100)
Rats & moles	15 (93.8)	1 (6.2)	16 (100)
N/A	19 (20.2)	75 (79.8)	94 (100)
**Total**	52 (34.9)	97 (65.1)	149 (100)
Environment free of contaminants			
Yes	3 (17.6)	14 (82.4)	17 (100)
No	49 (37.1)	83 (62.9)	132 (100)
**Total**	52 (34.9)	97 (65.1)	149 (100)

This variable was assessed in relation to food contamination, and a significant association was observed (*χ*^2^_2, 0.05_ = 37.270, *P*<.001). Those who had access to a modern toilet had a higher occurrence of food contamination (29%) compared with those who had access to a latrine (11.4%) (Table 2). Accessibility of running water around the toilet facility was thus negatively associated with food contamination (x^2^_2, 0.05_ = 36.046, *P*<.001), whereby a higher occurrence of food contamination was observed among those who did not have running water (30%) compared with those who had running water (15.9%) (Table 2).

Open area dumping was the most common method (62.4%) used by vendors to dispose waste. The relationship between waste disposal and food contamination was, however, not statistically significant (*χ*^2^_2, 0.05_ = 1.369, *P*=.504) (Table 1). Food contamination was highest among vendors who used municipal containers for waste disposal (40.4%). Presence of pests/rodents was significantly (*P*<.001) associated with food contamination, with vendors who reported presence of pests/rodents having a 5.9-fold risk (OR 5.921; 95% CI, 2.831–12.383) of having contaminated food (Table 1). The type of pests or rodents on-site was observed to have a significant association with food contamination (*χ*^2^_2, 0.05_ = 35.489, *P*<.001), with vendors who reported presence of both rats and moles having the highest occurrence of food contamination (93.8%) compared with those who reported rats only (46.2%) (Table 2).

Raw sewage lines, dust, flies, and vehicle fumes were some of the potential environmental contaminants reported by the vendors. The relationship between environmental contaminants and food contamination was not statistically significant (OR 0.363; 95% CI, 0.099–1.327; *P*=.113). However, those reporting the presence of such contaminants around their vending site had a higher occurrence of food contamination (37.1%) compared with those who did not (17.6%) (Table 2).

Some food-handling practices were significantly associated with food contamination. Access to fresh running water for hand washing while preparing food was negatively associated with food contamination (*P*<.005), whereby a decreased risk of having contaminated food was observed if one had access to fresh running water (OR 0.355; 95% CI, 0.177–0.713) (Table 3). The occurrence of food contamination was lower among those who had access to fresh running water for hand washing (24.1%) compared with those who did not (47.1%) (Table 4). Hand washing before handling food items was also negatively associated with food contamination (*P*<.005), whereby there was a decreased risk of contaminated food if vendors practised hand washing before handling food items (OR 0.018; 95% CI, 0.006–0.051) (Table 3). There was a lower occurrence of food contamination among those who washed hands before handling food items (10.8%) compared with those who did not (87.2%) (Table 4). Hand washing after handling raw food items was practised by only 13.4% (20) of the vendors. This variable was however not significantly associated with food contamination (OR 1.288; 95% CI, 0.490–3.383; *P*=.607) (Table 3).

**TABLE 3.** Bivariate Analysis of Food-Handling Practices in Relation to Occurrence of Food Contamination

Food-Handling Practices	Chi-square	df	*P* Value	OR	Lower CI	Upper CI
Access to fresh running water for hand washing while preparing food	8.711	1	.003^*^	0.355	0.177	0.713
Hand washing before handling food items	82.768	1	<.001^*^	0.018	0.006	0.051
Hand washing after handling raw food items	0.265	1	.607	1.288	0.490	3.383
Method of hand washing	60.657	3	<.001^*^	…	NA	NA
Acquisition of skills	1.317	3	.725	…	NA	NA
Medical check-up	4.216	3	.239	…	NA	NA
Use of an apron	21.296	1	<.001^*^	0.190	0.091	0.394
Use of a head cover	2.994	1	.084	0.531	0.258	1.093
Storage of leftover food	4.431	2	.109	…	NA	NA
Serving of food	0.755	3	.860	…	NA	NA

Abbreviations: CI, confidence interval; df, degrees of freedom; OR, odds ratio. Ellipses indicate the OR could not be calculated due to multiple responses to the variable.

* Variables significant at the 5% level.

**TABLE 4.** Occurrence of Food Contamination in Relation to Food-Handling Practices

	Food Contamination		
Food-Handling Practices	Yes No. (%)	No No. (%)	Total No. (%)
Access to fresh running water for hand washing while preparing food			
Yes	19 (24.1)	60 (75.9)	79 (100)
No	33 (47.1)	37 (52.9)	70 (100)
**Total**	52 (34.9)	97 (65.1)	149 (100)
Hand washing before handling food items			
Yes	11 (10.8)	91 (89.2)	102 (100)
No	41 (87.2)	6 (12.8)	47 (100)
**Total**	52 (34.9)	97 (65.1)	149 (100)
Hand washing after handling raw food items			
Yes	8 (40.0)	12 (60.0)	20 (100)
No	44 (34.1)	85 (65.9)	129 (100)
**Total**	52 (34.9)	97 (65.1)	149 (100)
Medical check-ups			
< 3 months apart	2 (20.0)	8 (80.0)	10 (100)
Every 3 months	6 (26.1)	17 (73.9)	23 (100)
> 3 months apart	17 (47.2)	19 (52.8)	36 (100)
Never	27 (33.8)	53 (66.2)	80 (100)
**Total**	52 (34.9)	97 (65.1)	149 (100)
Use of an apron			
Yes	16 (19.0)	68 (81.0)	84 (100)
No	36 (55.4)	29 (44.6)	65 (100)
**Total**	52 (34.9%)	97 (65.1)	149 (100)
Use of a head cover			
Yes	15 (26.3)	42 (73.7)	57 (100)
No	37 (40.2)	55 (59.8)	92 (100)
**Total**	52 (34.9)	97 (65.1)	149 (100)
Storage of leftover food			
Fridge	5 (18.5)	22 (81.5)	27 (100)
Cupboard	36 (40.4)	53 (59.6)	89 (100)
Other	11 (33.3)	22 (66.7)	33 (100)
**Total**	52 (34.9)	97 (65.1)	149 (100)
Serving of food			
Plastic bag	34 (33.7)	67 (66.3)	101 (100)
Both newspaper and plastic	5 (35.7)	9 (64.3)	14 (100)
Plastic or plate	4 (42.9)	9 (69.2)	13 (100)
Other	9 (30.8)	12 (57.1)	21 (100)
**Total**	52 (34.9)	97 (65.1)	149 (100)

The method of hand washing was also assessed in relation to food contamination. The highest occurrence of food contamination (85%) was among vendors who did not wash their hands at all, either before handling food items or after handling raw food items. This variable was significantly associated with food contamination (*χ*^2^_3, 0.05_ = 60.657, *P*<.001) (Table 3). In terms of acquisition of knowledge on food preparation, a majority of vendors (63.8%) had acquired food preparation skills through observation, and only 3.4% had been formally trained. On examining this variable in relation to food contamination, there was no significant association (*χ*^2^_3, 0.05_ = 1.371, *P*=.725) (Table 3).

Although the relationship between having a medical examination (check-up) and food contamination was not significant (*x*^2^_3, 0.05_ = 4.216, *P*=.239) (Table 3), the occurrence of food contamination was highest among vendors who had regular check-ups more than 3 months apart (47.2%) and least among those who had their medical check-ups less than 3 months apart (20%) (Table 4).

Use of protective clothing while vending food was also assessed in relation to food contamination. There was a significant association between use of an apron and food contamination (*P*<.001), whereby a decreased risk was observed if one wore an apron (OR 0.190; 95% CI, 0.091–0.394) (Table 3). Although use of a cap to cover hair was not significantly associated with food contamination (OR 0.531; 95% CI, 0.258–1.093; *P*=.084), there was a higher occurrence of food contamination among the respondents who did not use a cap (40.2%) compared with those who did use a cap while vending food (26.3%) (Table 4).

Storage of leftover food was also assessed in relation to food contamination. It was observed that a majority of vendors (59.7%) stored leftover food in a cupboard, and only 18.1% stored it in a refrigerator. Although this variable was not significantly associated with food contamination (*χ*^2^_2, 0.05_ = 4.431, *P*=.109), there was a higher occurrence of food contamination among respondents who stored leftover food in a cupboard (40.4%) compared with those who either stored the food in a refrigerator (18.5%) or those who did ‘other’ and either consumed leftovers with family, gave it to friends, or had no leftover food (33.3%) (Table 4). In terms of serving food, a majority (67.8%) used a plastic bag since they sold takeaway food. There was no significant association between the way food was served and food contamination (*χ*^2^_3, 0.05_ = 0.755, *P*=.860). However, a higher occurrence of food contamination (42.9%) was observed among vendors who used a plastic bag or plates, and the least occurrence of food contamination (30.8%) was seen among those in the ‘other’ category, who mainly used labelled paper bags distributed by manufacturers for serving sausages (Table 4).

## DISCUSSION

According to Baluka and colleagues, environmental hygiene is important for food safety and necessary to support safe food handling and hygiene by employees.^13^ The present study explored the relationship between various environmental factors and food contamination, and a significant (*P*<.05) association was observed for some of them. A negative association was observed between access to a toilet facility and food contamination (*P*<.001), whereby a decreased risk of occurrence of food contamination was observed if one had access to a toilet facility (OR 0.095; 95% CI, 0.03–0.227). Similarly, Idowu and Rowland^14^ reported that vending sites usually lack basic facilities such as toilets and hand washing facilities, since nearness to customers is the primary target of street food vendors. These conditions enhance the incidence of foodborne illnesses and transmission of diseases.

**Figure:**
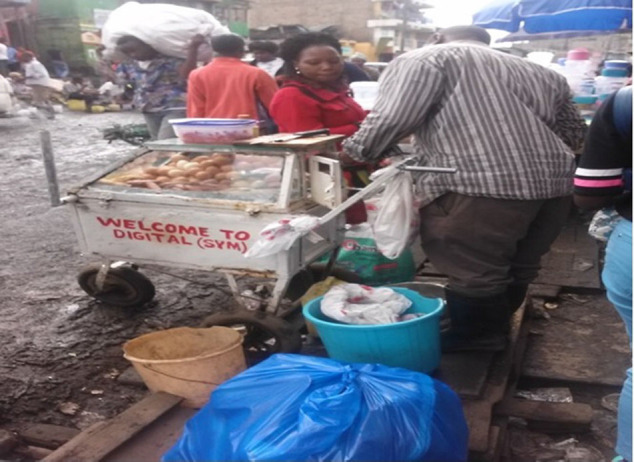
Sludge formed by wastewater from a burst sewer.

©2015 Nicholas Lagat

The main type of toilet facility observed in the 2 study areas was the modern toilet (46.3%). The greatest challenge that was observed with this type of toilet facility was the inadequate sewerage system. In 1 study area, wastewater flowed along the street as a result of a burst sewer. The vendors along the street continued selling food, oblivious to the hazard posed by the burst sewer. This provided a favorable environment for flies and other types of vectors of transmission of pathogens. This may have contributed to the higher occurrence of food contamination (29%) among vendors who had access to a modern toilet.

Accessibility of running water around the toilet facility for hand washing was negatively associated with food contamination in this study (*χ*^2^_2, 0.05_ = 36.046 *P*<.001). A higher occurrence of food contamination was observed among vendors who had no access to running water (30%) compared with those who had access to running water (15.9%). This implies that water is a basic necessity that ensures better personal hygiene, which in return serves to reduce the potential for food contamination. Other studies have observed that food that has been properly prepared can become contaminated when handled by people with unwashed hands and that poor access to hand washing water can be a source of bacterial contaminants for food.^15,16^

**Figure:**
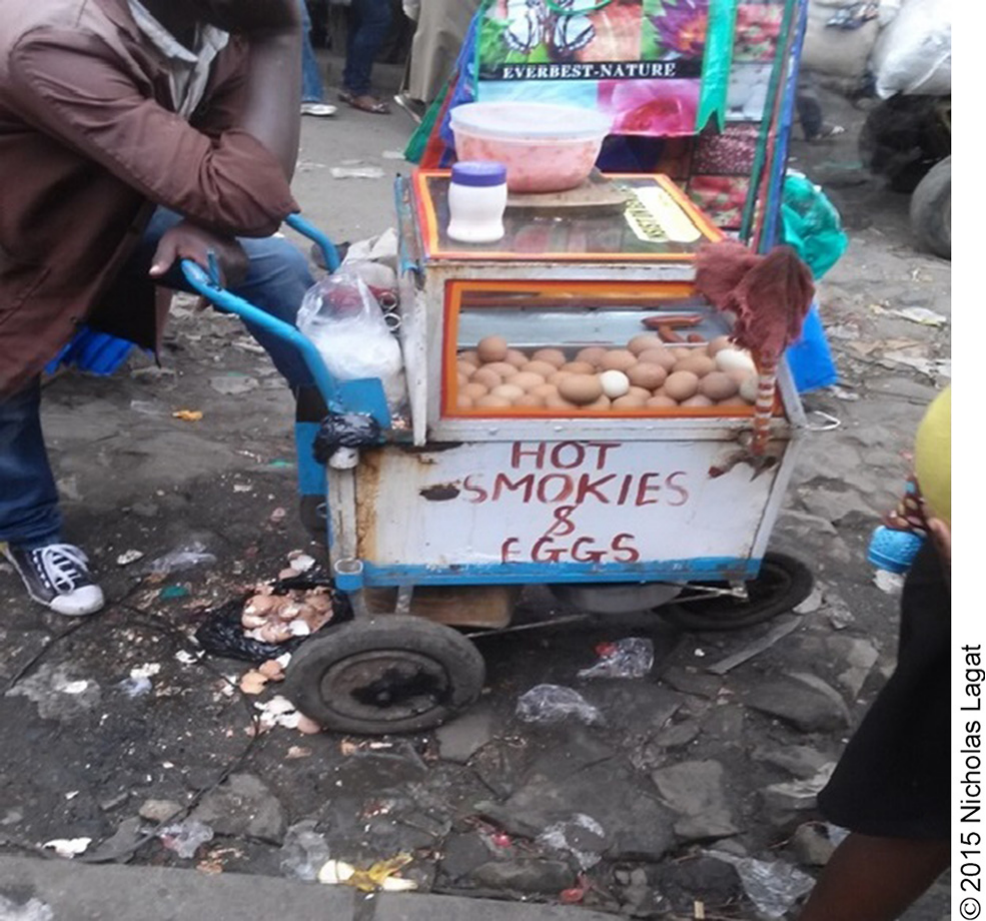
Waste disposed on the ground by a vendor.

©2015 Nicholas Lagat

A majority (62.4%) of the vendors in this study practised open area dumping to dispose of waste. The open area dumping sites were, however, located at a distance away from most vending sites, and hence the vendors stored their waste in plastic bags within the site, then disposed of it later. Although the relationship between waste disposal and food contamination was not statistically significant (*P*>.05), vendors who used municipal containers had the highest occur-rence of food contamination (40.4%), probably because these containers were filled beyond capacity and attracted flies that could serve as vehicles of transmission of food contaminants. These vendors would then resort to discarding waste on the ground. These findings agreed with observations made in a study in Uganda where focus group discussions revealed that the municipal council containers were not regularly emptied, therefore in most cases, they were also overflowing. This created a dirty environment that compromised sanitation, became a habitat for rodents and a breeding point for flies, and promoted the growth of microorganisms.^17^

This study found that the relationship between environmental contaminants and food contamination was not statistically significant (*P*>.05). Nevertheless, vendors reporting the presence of contaminants such as flies, dust, insects, rodents, and sludge around the vending site had a higher occurrence of food contamination (37.1%) compared with those who thought that the environment had none of these contaminants (17.6%). According to Muyanja, dust carries many microbes that may be pathogenic if left to settle onto prepared foods.^17^ The presence of rats and moles, however, was significantly associated with food contamination (*P*<.05). Vendors who reported the presence of these rodents had a higher occurrence of food contamination (60%) compared with those who did not (20.2%). The rodents may have found an environment that was conducive for breeding as a result of the poor methods of waste disposal and may have served as agents of transmission of contaminants onto prepared foods.

This study observed that access to fresh running water where food was being prepared was negatively associated with food contamination (*P*<.005), whereby a decreased risk of having contaminated food was observed if there was access to fresh running water (OR 0.355; 95% CI, 0.177–0.713). A study in Malawi similarly observed that poor access to fresh running water provides harbour to faecal bacteria that can serve as a source of bacterial contaminants in food.^15^ Hand washing before handling food items was also negatively associated with food contamination (*P*<.005), whereby there was a decreased risk of having contaminated food if one practised hand washing before handling food items (OR 0.018; 95% CI, 0.006–0.051). This suggests that observing personal hygiene can help in reduction of food contamination. The findings of this study agree with a study in Ethiopia that observed that vendors with poor personal hygiene had a 4-fold risk of having contaminated food as compared with those who had good personal hygiene.^18^

Contrary to what has been found in the available literature, food contamination was higher among vendors who said they washed their hands after handling raw food items (40%) compared with those who said they did not (34.1%). This finding may have been a result of use of recycled water for hand washing. The vendors cleaned the raw food items using water placed in a container, then used the same water to clean the knives used for cutting and also to wash their hands. This presented a potential risk of cross-contamination as a result of using recycled water for hand-washing. On the other hand, those who did not wash their hands after handling raw food items were mainly vendors who sold takeaway food items such as boiled eggs and sausages that were served with raw vegetables. The raw vegetables were mainly tomatoes and onions which they had already prepared at home. In the event that it was necessary for them to prepare the vegetables on-site, they claimed that they used a plastic bag to wrap their hands while serving to prevent direct contact with prepared foods. Through observation, it was noted that this practice was used by most of these vendors.

The method of hand washing was significantly associated with food contamination (*χ*^2^_3, 0.05_ = 60.657, *P*<.001). The highest occurrence (85%) was among vendors who neither washed their hands before handling food items nor after handling raw food items. The vendors who washed their hands using soap and running water had no occurrence (0%) of food contamination. Occurrence of food contamination was 17.2% among vendors who used plain water placed in a container and 16.7% among those who used soap and water placed in a container. This may imply that proper hand washing skills reduces the potential for occurrence of food contamination.

These findings are consistent with findings of Todd and colleagues, who reported that several foodborne disease outbreaks were a result of poor handling practices, such as cross-contamination between raw and cooked products and poor personal hygiene of food handlers, such as failure to wash hands.^19^

We found a significant negative association between use of an apron and food contamination (*P*<.05). Vendors who wore an apron had a lower occurrence of food contamination (19%) compared with those who did not (55.4%). On the other hand, although use of a head cover was not significantly associated with food contamination (*P*>.05), there was a higher occurrence of food contamination among the vendors who did not use a head cover (40.2%) compared with those who did (26.3%). Similarly, in a study in Togo, failure to wear aprons and caps was observed to be the likely causative factor for contamination of food samples.^20^ These findings suggest that use of protective clothing is necessary to reduce the likelihood of food contamination.

In this study, storage of leftover food was not significantly associated with food contamination (*P*>.05). However, the study explored only the method of storage and not the duration of storage. This could be the reason why our findings differ from those of a study in Ethiopia that observed that storage of leftover street-vended foods for more than a day was a risk factor for contamination (*P*<.05).^18^ Findings from this study showed no significant association between the way food was served and food contamination (*χ*^2^_3, 0.05_ = 0.755, *P*=.860). However, a higher occurrence of food contamination was observed among vendors who used a plastic bag or plates (42.9%), and the least amount of contamination was found among vendors who used labelled paper bags normally distributed by manufacturers (30.8%).

The labelled paper bags were mostly used for the sale of sausages or ‘smokies’, which is a type of sausage. Newspapers were used for wrapping food only after it was first wrapped inside a plastic bag. The type of foods that were mainly served in this manner were fried chips and fish. The plastic bags, however, were poorly stored, as most vendors kept them in the open, which posed a risk of contamination from the environment. Some vendors also blew air into the plastic bags before serving the food, and others would cut the bags into small pieces so as to avoid ‘misuse’. The small pieces of plastic bags would then be used to serve foods such as boiled eggs, with some vendors charging a higher price if a consumer needed to have the food completely wrapped. These kinds of practices may have led to the higher occur-rence of food contamination among vendors who used plastic bags or plates to serve food, since the plates were, on the other hand, cleaned using recycled water. These findings were consistent with observations made in a study in Haiti where bags and plates were identified to be some of the possible sources of food contamination.^21^ According to Barro and colleagues, plastic bags are usually contaminated by the food handlers, as pathogens may invade the interior surfaces of the bags during packaging of the food due to poor handling practices.^22^

Some environmental factors and food-handling practices did not show any significant relationship with food contamination in this study. This may imply that there was no risk of food contamination if direct contact was not made with food. Similar findings were observed in a study in Accra that found that environmental hygiene and the vendor's appearance did not show any significant relationship with the levels of contamination.^23^

## CONCLUSION

Access to a toilet facility and availability of running water within the toilet facility were the environmental factors found likely to decrease the risk of food contamination. On the other hand, the presence of pests and rodents around the vending sites was likely to increase the risk of food contamination. Access to fresh running water while preparing food, hand washing before handling food items, and use of protective clothing (apron) were the food-handling practices likely to decrease the risk of food contamination. There is a need for market vendors to be provided with more basic amenities as well as with sensitisation on pest control to reduce the potential for food contamination.
